# Post-operative Non-sustained Ventricular Tachycardia in a COVID-19 Positive Patient After Ketamine and Dexmedetomidine Infusion

**DOI:** 10.7759/cureus.20300

**Published:** 2021-12-09

**Authors:** Vandana Sharma, Muhammad Sarwar, Evin Koleini

**Affiliations:** 1 Anesthesiology, State University of New York Upstate Medical University, Syracuse, USA

**Keywords:** atrial fibrillation, ventricular tachycardia, dexmedetomidine, cardiac, pacemaker, covid-19

## Abstract

We present a case of an elderly, 75-year-old gentleman with COVID-19 infection and a permanent pacemaker in place, presenting for elective procedure, under monitored anesthesia care with dexmedetomidine. In the postoperative period, while still in the Post Anesthesia Care Unit (PACU), the patient became unresponsive and was found to have episodes of arrhythmia. The patient was managed in the PACU by the anesthesia team. To date, our report is the only one addressing cardiac complications in elderly patients with preexisting cardiac comorbidity, in the immediate perioperative period.

## Introduction

Since the end of 2019, the world has been facing a pandemic of coronavirus disease-2019 (COVID-19). To date, there have been more than 150 million cases worldwide with a number of deaths related to COVID-19 rapidly reaching 3 million per Reuters COVID-19 tracker [1]. COVID-19 is caused by the acute respiratory syndrome coronavirus 2 (SARS-CoV-2) [2]. Although primary presenting signs of COVID-19 infection remain respiratory, cardiovascular complications from this infection are contributing to high mortality of patients, especially with preexisting cardiac conditions [2]. This case report will provide an insight into the cardiac implications of COVID-19 that an anesthesiologist might encounter while caring for these patients.

## Case presentation

A 75-year-old, morbidly obese Caucasian male presented to the operating room for wide local excision of the left submandibular neck mass. The patient had a past medical history of obstructive sleep apnea, mild pulmonary hypertension, chronic obstructive pulmonary disease (COPD) (one pack per day smoker who abstained for one year), atrial fibrillation, and sick sinus syndrome requiring permanent VVI pacemaker implantation. The pacemaker was interrogated before surgery and the patient was not found to be pacemaker dependent. His preoperative blood work was otherwise normal. The patient obtained a dobutamine stress test one week prior which was negative. The patient tested positive for COVID-19 three days prior to surgery on May 26, 2020. He remained asymptomatic at his baseline respiratory status. Given the urgent need for obtaining a diagnosis with an increasing neck mass, the patient elected to proceed with the surgery after a discussion with the surgical and anesthesia teams.

Anesthetic planning was done after a thoughtful discussion of risks and benefits with the patient and the surgical team. We decided to proceed with monitored anesthesia care, given the superficial location of the mass and multiple risk factors for postoperative cardiac and pulmonary adverse events. The patient was pretreated with IV midazolam 1 mg and glycopyrrolate 0.2 mg in the preoperative area. The intraoperative anesthetic plan was then carried out using IV infusion of dexmedetomidine at 0.8 mcg/kg/hr and intermittent boluses of IV ketamine 10 mg (total dose 50 mg used throughout the case). No opioids were used during the entire case. The patient maintained spontaneous breathing with supplemental oxygen using nasal cannula at 3 L/min. He tolerated the procedure well and was transferred to the recovery room uneventfully.

**Figure 1:**
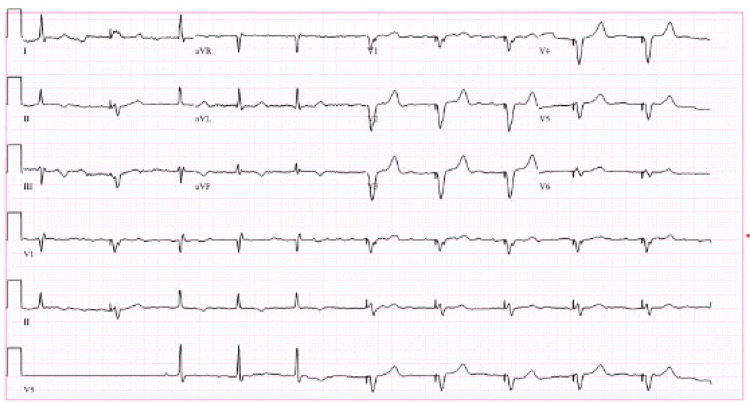
EKG in PACU that demonstrated a paced rhythm but NSVT could not be captured at this point PACU - Post Anesthesia Care Unit, NSVT - non-sustained ventricular tachycardia

Shortly after his arrival in the recovery room, he became unresponsive to verbal commands. This was accompanied by hypotension with a drop of blood pressure from 135/66 to 79/28 and a feeble pulse. The peripheral pulse oximeter failed to show oxygen saturation, and it was unclear if he was breathing spontaneously. Therefore, airway management was immediately taken over by the anesthesiologist using a bag and mask ventilation, and help was called. The patient regained consciousness within less than a minute with improvement in blood pressure and spontaneous breathing. The pulse oximeter showed 100% oxygen saturation once hemodynamics were restored. The patient never lost peripheral pulses and CPR was not performed. He was given a fluid bolus while preparing vasopressors, but the blood pressure returned to normal spontaneously without the need for vasopressors. The patient subsequently became more alert and was able to respond to verbal commands appropriately. During this time, the attached 5-lead EKG monitor showed inconsistent runs of non-sustained ventricular tachycardia (NSVT) followed by paced rhythm, but no changes in neurological or hemodynamic status. A 12-lead EKG was obtained once the patient became stable which showed paced rhythm, but NSVT could not be captured (Figure 1).

This episode was shortly followed by another similar but brief episode of unresponsiveness accompanied by hypotension that subsided without intervention within a short period of time as previously. The patient continued to have multiple intermittent runs of NSVT and defibrillator pads were applied anticipating further conduction changes. At this time, we also obtained emergent labs using point of care testing, which included cardiac enzyme markers and venous blood gas. The lab results were normal except for hypocalcemia (corrected calcium level of 8.0), elevated inflammatory markers, and D-dimer activity. Electrolytes were corrected using an infusion of calcium chloride 800 mg IV and magnesium sulfate 2 g IV was also given prophylactically. The patient was kept under close observation during this time, and cardiology was immediately consulted. Due to the unknown etiology of the arrhythmia, our team initially held off from starting Amiodarone prior to obtaining cardiology consultation. The patient improved over the next several hours, was completely asymptomatic the next day, and was discharged home. A repeat 2-D echocardiogram was obtained six months later which demonstrated baseline function from the echocardiogram preoperatively; an ejection fraction greater than 55% and no structural cardiac compromise.

## Discussion

Upon a thorough analysis of events, we believe the episode of unconsciousness was secondary to transient cardiac arrhythmia resulting in global hypoperfusion in our COVID-19 positive patient with multiple cardiopulmonary co-morbidities. In general, cardiovascular complications attributed to COVID-19 infection range from myocardial injury, myocarditis, myocardial infarction, acute heart failure, cardiomyopathy, dysrhythmias, and venous thromboembolic events [3,4]. Manifestations of these complications are expected to be more likely in the presence of preexisting cardiac conditions [4,5]. Cardiac conduction abnormalities from COVID-19 range from sinus tachycardia, atrial fibrillation, bradycardia, ST-segment changes, T-wave changes, QT interval prolongation, and ventricular tachycardia [6].

Experiences from previous viral infections have demonstrated the fact that myocardial cellular injury is a result of a combination of viral entry into the myocytes with a resulting immune response that can lead to myocardial necrosis [7]. Clinical symptoms can result from this injury within a few days [7].

Although a variety of arrhythmias associated with COVID-19 infections have been reported, the most commonly reported is tachycardia, presenting as palpitations [8]. This could be the result of hypoperfusion, fever, hypoxia, anxiety, etc. [8]. A study of 138 patients by Wang et al. concluded that arrhythmias were observed in 17% of patients hospitalized with COVID-19 and among the ICU patients, 44% exhibited various arrhythmias [9]. A number of reasons have been proposed for arrhythmias, including inflammatory stress metabolic abnormalities [10]. A myocardial injury should be strongly considered if arrhythmias are associated with rising troponins [10].

The effects of medications on these patients cannot be ignored. Dexmedetomidine is commonly used in the ICUs and as an adjunct to anesthesia. Dexmedetomidine is a medication with anxiolytic, sedative, analgesic sparing, and sympatholytic effects via its alpha 2 adrenoceptor agonist. Dexmedetomidine is known to cause transient hypertension, bradycardia, and hypotension [11], and has not been shown to have a cardioprotective role in patients with preexisting cardiac history [11]. Interestingly, although these hemodynamic effects are well documented, an overall organ-protective effect of dexmedetomidine in COVID-19 has been reported due to its role in inflammatory modulation and antioxidative effects [12-15].

None of the reports available in the literature address any cases of arrhythmias in a patient in the perioperative period and under general anesthesia and specifically in patients who had a cardiac implantable electrical device (CIED) in place. To date, our report is the first one to address COVID-19 cardiac complications in the perioperative period in this very specific population of patients.

## Conclusions

COVID-19 poses a set of challenges that are relatively new in many ways from what clinicians have encountered in the past. More work needs to be done to ascertain the cardiac manifestations of anesthetics in patients suffering from COVID-19. In conclusion, although our report does not definitely link dexmedetomidine or any other drug to our patient’s arrhythmias, anesthetic management of a patient with COVID-19 infection and preexisting cardiac comorbidities should encompass management of cardiac events in the perioperative period. Careful choice of medications for these patients cannot be stressed enough. Given the altered physiology of this population of patients, extra vigilance is warranted.

## References

[1] (2021). Reuters COVID-19 Tracker. https://graphics.reuters.com/world-coronavirus-tracker-and-maps/countries-and-territories/united-states/.

[2] Nishiga M, Wang DW, Han Y, Lewis DB, Wu JC (2020). COVID-19 and cardiovascular disease: from basic mechanisms to clinical perspectives. Nat Rev Cardiol.

[3] Long B, Brady WJ, Koyfman A, Gottlieb M (2020). Cardiovascular complications in COVID-19. Am J Emerg Med.

[4] Li B, Jin X, Zhang T (2020). Comparison of cardiovascular metabolic characteristics and impact on COVID-19 and MERS. Eur J Prev Cardiol.

[5] Bansal M (2020). Cardiovascular disease and COVID-19. Diabetes Metab Syndr.

[6] Long B, Brady WJ, Bridwell RE (2021). Electrocardiographic manifestations of COVID-19. Am J Emerg Med.

[7] Cooper LT Jr (2009). Myocarditis. N Engl J Med.

[8] Chen Q, Xu L, Dai Y (2020). Cardiovascular manifestations in severe and critical patients with COVID-19. Clin Cardiol.

[9] Wang Y, Wang Z, Tse G (2020). Cardiac arrhythmias in patients with COVID-19. J Arrhythm.

[10] Hendren NS, Drazner MH, Bozkurt B, Cooper LT Jr (2020). Description and proposed management of the acute COVID-19 cardiovascular syndrome. Circulation.

[11] Gertler R, Brown HC, Mitchell DH, Silvius EN (2001). Dexmedetomidine: a novel sedative-analgesic agent. Proc (Bayl Univ Med Cent).

[12] Tosun Z, Baktir M, Kahraman HC, Baskol G, Guler G, Boyaci A (2013). Does dexmedetomidine provide cardioprotection in coronary artery bypass grafting with cardiopulmonary bypass? A pilot study. J Cardiothorac Vasc Anesth.

[13] Zhao H, Davies R, Ma D (2021). Potential therapeutic value of dexmedetomidine in COVID-19 patients admitted to ICU. Br J Anaesth.

[14] Jain A, Lamperti M, Doyle DJ (2021). Dexmedetomidine: another arrow in the quiver to fight COVID-19 in intensive care units. Br J Anaesth.

[15] Turan A, Duncan A, Leung S (2020). Dexmedetomidine for reduction of atrial fibrillation and delirium after cardiac surgery (DECADE): a randomised placebo-controlled trial. Lancet.

